# Short and sweet: an analysis of the length of parasite species names

**DOI:** 10.1007/s11230-022-10058-0

**Published:** 2022-08-03

**Authors:** Robert Poulin, Daniela de Angeli Dutra, Bronwen Presswell

**Affiliations:** Department of Zoology, University of Otago, PO Box 56, Dunedin, New Zealand

## Abstract

In its advice to taxonomists, the International Commission on Zoological Nomenclature (ICZN) recommends that scientific species names should be compact, memorable, and easy to pronounce. Here, using a dataset of over 3000 species of parasitic helminths described in the past two decades, we investigate trends in the length of Latin specific names (=epithets) chosen by taxonomists. Our results reveal no significant temporal change in the length of species epithets as a function of year of description, with annual averages fluctuating around the overall average length of just over 9 letters. We also found that lengths of species epithets did not differ among the various host taxa from which the parasites were recovered, however acanthocephalan species have been given longer species epithets than other helminth taxa. Finally, although species epithets were shorter than genus names for three-quarters of the species in our dataset, we detected no relationship between the length of species epithets and that of genus names across all species included, i.e., there was no evidence that shorter species epithets are chosen to compensate for long genus names. We conclude by encouraging parasite taxonomists to follow the recommendations of the ICZN and choose species epithets that are, as much as possible, compact and easy to remember, pronounce and spell.

**Supplementary Information:**

The online version contains supplementary material available at 10.1007/s11230-022-10058-0.

## Introduction

From the 18^th^ century when Carl Linnaeus proposed his taxonomic classification scheme, to the central place it now occupies in the International Commission on Zoological Nomenclature’s (ICZN) framework (https://www.iczn.org/), the Latin binomial system is a cornerstone of taxonomy and all efforts to inventory Earth’s biodiversity. For ease of use, the ICZN and various commentators (e.g., Šlapeta 2013) recommend names that are compact, memorable, and/or easy to pronounce. However, many species names pose challenges to both scientists and lay people who must pronounce, remember or spell them. How have taxonomists, including parasite taxonomists, followed the above recommendations when naming new species after their initial discovery?

There is much variation in the length of species names. According to Wikipedia (https://en.wikipedia.org/wiki/List_of_short_species_names), the shortest binomial species names (genus and species names combined) are 4 letters long, including the bat *Ia io* Thomas 1902 from tropical Asia. Species names cannot be any shorter than this. Also according to Wikipedia (https://en.wikipedia.org/wiki/List_of_long_species_names), the bacteria *Myxococcus llanfairpwllgwyngyllgogerychwyrndrobwllllantysiliogogogochensis* Chambers et al. 2020 (73 letters for genus and species names combined) is the longest binomial species name as of 2022. It is named after the site in Wales where it was discovered, which has one of the longest place names in the world. It is hardly easy to spell or pronounce, let alone remember. According to the same source, the parasite with the longest name is the trematode *Epithelionematobothrium mulloidichthydis* Yamaguti, 1970, with 39 letters; the nematode *Hysterothylacium deardorffoverstreetorum* Knoff et al. 2012 also has a name with 39 letters. These are both a challenge to write or say out loud.

When choosing a name for a new species, taxonomists are faced with a compromise. On the one hand, short and simple names should be more appealing because they will be easy to remember. On the other hand, longer names may be necessary to fully capture who or what they want to honour or the information they want to convey about the species (where it was found, what it looks like, what host species it infects). It remains unclear whether patterns in name lengths emerge from the vast number of parasitic helminth species named to date. Do species names differ in length among different taxa of parasites, or among the host taxa from which they are found, possibly reflecting different traditions or preferences among the taxonomists working on those taxa? For each major parasite taxon, a small number of prolific researchers account for the vast majority of new species described and named (Poulin & Presswell 2022), therefore we might expect their influence to be reflected in the names chosen. Also, has the average length of new species names changed over time, perhaps reflecting a growing preference for shorter names? Finally, are shorter species epithets more frequently chosen for species belonging to genera with long names, as an effort (perhaps subconscious) to keep the full binomial name within reasonable length?

Here, we address the above questions using a large dataset on the species names of helminth parasites described since the year 2000. We focus exclusively on species epithets, and not on genus names or full binomial names, since many genera have been named well before the starting year of our dataset. In other words, except when new genera are erected, the morphology and genetics of a new species determine what existing genus it falls into; only the species epithet gets chosen from scratch by the authors of the species description.

## Methods

We used the dataset compiled by Poulin et al. (2022), which comprises information on each new species description of trematodes, cestodes, monogeneans, nematodes, and acanthocephalans published between 2000 and 2020, inclusively, in the following 8 journals: *Acta Parasitologica* (data from 2000–2005 missing for this journal), *Comparative Parasitology*, *Folia Parasitologica*, *Journal of Helminthology*, *Journal of Parasitology*, *Parasitology International*, *Parasitology Research*, and *Systematic Parasitology*. We updated this dataset with data from new species described in the same 8 journals in 2021. Although helminth descriptions are also published in other journals, these 8 journals capture a large proportion of published descriptions, and provide a large enough sample for the present analysis. The full dataset is provided as Supplementary Information.

For each species description, in addition to the Latin binomial name of the new species, the dataset includes the following information: (i) the higher taxon to which the parasite belongs (trematodes, cestodes, monogeneans, nematodes, or acanthocephalans); (ii) the host taxon it parasitises (invertebrates, mammals, birds, reptiles, amphibians, or fish including elasmobranchs); (iii) the number of letters in both the genus name and species epithet; (iv) the year of publication; and (v) the journal in which it was published.

Our analysis tested for taxonomic or temporal patterns in the length of parasite species epithets, as well as for a relationship between the lengths of genus names and species epithets. For this, we used the length (no. letters) of species epithets as response variable in a generalized linear mixed model (GLMM) with Poisson distribution, using the *lme4* package (Bates et al. 2015) in the R computing environment (R Core Team 2022). The fixed factors or predictors were the length of the genus name, the parasite’s higher taxon (5 levels: trematodes, cestodes, monogeneans, nematodes, and acanthocephalans), the host taxon (6 levels: invertebrates, fish, amphibians, reptiles, birds and mammals), and the year of publication (2000 to 2021; ordered variable). For the two categorical factors, based on earlier pairwise analyses, ‘acanthocephalan’ was chosen as the reference level (included in the intercept) for parasite taxa because it tended to differ from other taxa, whereas ‘amphibian’ was chosen arbitrarily as the reference level for host taxa as no difference was seen among host taxa. Interactions were left out of the model, as the number of possible combinations was too large for meaningful interpretation. The journal in which the species was described was included as a random factor, to account for non-independence among species descriptions and possible (though unlikely) editorial pressures creating consistent inter-journal differences in the length of species epithets.

## Results

**Fig. 1:**
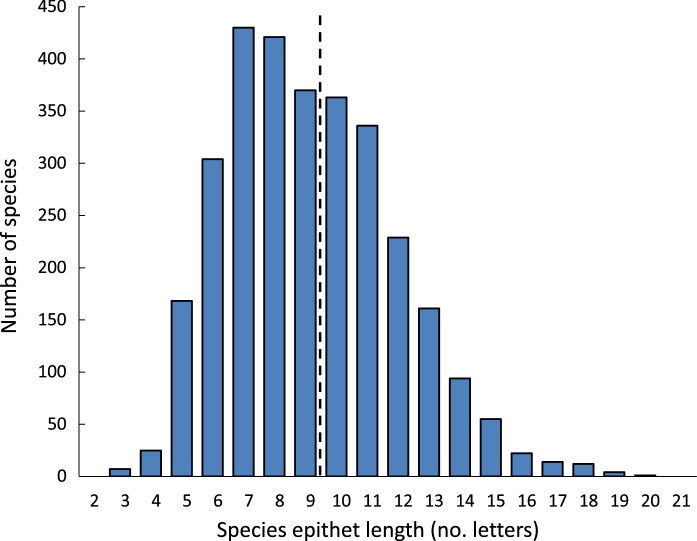
Frequency distribution of the lengths of species epithets among 3016 species of helminth parasites described between 2000 and 2021 inclusively. The broken line indicates the average length.

Our 22-year dataset (2000–2021 inclusively) comprised 3016 species names, with monogeneans and nematodes accounting for the majority of species (Table 1). The lengths of species epithets followed an approximate Poisson distribution (Fig. 1), with an overall average length of 9.2 letters (range 3 to 20). The longest epithets are far from easy to pronounce or spell (Table 2). Several species epithets in our dataset were used for more than one species. The most popular ones were the 7-letter name ‘*gibsoni*’ (used for 13 species), followed by ‘*brayi*’ (5-letters; used for 11 species) and ‘*vietnamensis*’ (12-letters; used for 9 species). These were treated as separate entries, because they were chosen by their authors independently of each other even if they have the same etymology (i.e., eponyms of the eminent taxonomists David Gibson and Rod Bray, and country of collection). The full dataset is provided as Supplementary Information.

**Table 1:** Average length (no. letters) of species epithets in the dataset broken down by parasite and host taxonomic groups. The number of species is given in parenthesis.

	Trematodes	Cestodes	Monogeneans	Nematodes	Acanthocephalans	TOTAL
Invertebrates	8.0 (1)	— (0)	— (0)	9.8 (75)	— (0)	9.8 (76)
Fish	8.9 (484)	8.7 (395)	9.2 (719)	9.8 (314)	10.3 (93)	9.2 (2005)
Amphibians	10.0 (22)	7.7 (3)	9.1 (15)	9.5 (95)	9.5 (8)	9.5 (143)
Reptiles	9.1 (51)	10.1 (19)	10.3 (6)	9.5 (157)	9.3 (6)	9.5 (239)
Birds	9.1 (103)	8.5 (36)	— (0)	8.6 (40)	9.9 (24)	9.0 (203)
Mammals	9.6 (43)	8.8 (73)	— (0)	9.0 (221)	8.3 (13)	9.0 (350)
TOTAL	9.0 (704)	8.7 (526)	9.2 (740)	9.4 (902)	10.0 (144)	9.2 (3016)

**Table 2:** Ten longest species epithets (based on number of letters) in our dataset

Species name	Higher taxon	Length of species epithet	Reference
*Rhabdias pseudosphaerocephala*	Nematode	20	Kuzmin et al. (2007)
*Hamatopeduncularia longiangusticirrata*	Monogenean	19	Soo & Tan (2021)
*Rhadinorhynchus dorsoventrospinosus*	Acanthocephalan	19	Amin et al. (2011)
*Paratrajectura longcementglandatus*	Acanthocephalan	19	Amin et al. (2018)
*Aenigmatrema undecimtentaculatum*	Trematode	19	Corner et al. (2020)
*Gyrocerviceanseris passamaquoddyensis*	Monogenean	18	Cone et al. (2010)
*Pseudocapillaria novaecaledoniensis*	Nematode	18	Moravec & Justine (2010)
*Bicentenariella puertopizarroensis*	Monogenean	18	Cruces et al. (2021)
*Neomultitestis aspidogastriformis*	Trematode	18	Bray & Cribb (2003)
*Ichthyofilaria novaecaledoniensis*	Nematode	18	Moravec & Justine (2009)

The GLMM results confirmed that the journal in which a species description was published accounted for a trivial proportion of variance in the length of species epithets (Table 3). The findings also indicate that the species epithets of acanthocephalans are longer than those of species in other taxa, with the difference being significant for trematodes, cestodes, and monogeneans but not quite for nematodes (Table 3). On average, epithets of acanthocephalan species were about one letter longer than those of species in other taxa (Table 1). In contrast, the results of the analysis revealed no significant variation in the length of species epithets among the host taxa from which the parasites were recovered.

**Table 3:** Results of the GLMM with length of the species epithet as the response variable, showing the effects of the main predictors. Significant effects shown in bold

Fixed factors	Estimate	Standard error	*z*-value	*p*-value
(intercept)	−0.030	2.095	−0.014	0.9885
Genus name length	−0.001	0.002	−0.170	0.8647
**Parasite taxon (cestodes)**	**−0.132**	**0.031**	**−4.317**	**<0.0001**
**Parasite taxon (monogeneans)**	**−0.080**	**0.029**	**−2.685**	**0.0073**
Parasite taxon (nematodes)	−0.057	0.030	−1.871	0.0614
**Parasite taxon (trematodes)**	**−0.098**	**0.030**	**−3.286**	**0.0010**
Host taxon (birds)	−0.037	0.037	−1.003	0.3157
Host taxon (fish)	−0.090	0.245	−0.369	0.7118
Host taxon (invertebrates)	0.030	0.046	0.648	0.5172
Host taxon (mammals)	−0.048	0.033	−1.461	0.1440
Host taxon (reptiles)	−0.001	0.034	−0.043	0.9659
Year of publication	0.001	0.001	1.123	0.2615

NB: acanthocephalans (parasite taxon) and amphibians (host taxon) are included in the intercept and serve as reference.

The percentage of the remaining variance accounted for by the random factor ‘Journal ID’ was <1%.

**Fig. 2:**
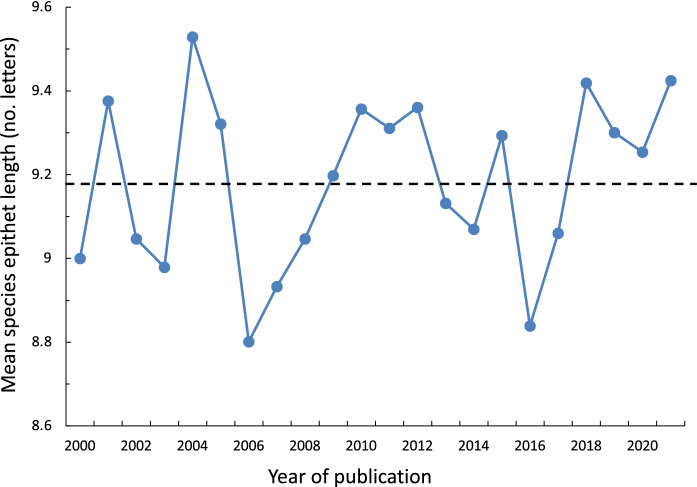
Annual average length of species epithets for helminth parasites described between 2000 and 2021. The broken line indicates the overall average length. The number of species on which the averages are based range from 85 (in 2002) to 178 (in 2007).

The analysis also uncovered no effect of year of publication on the length of species epithets (Table 3). The annual average length of species epithets has fluctuated slightly over time around the overall average (Fig. 2), showing no evidence of any clear and consistent temporal trend.

**Fig. 3:**
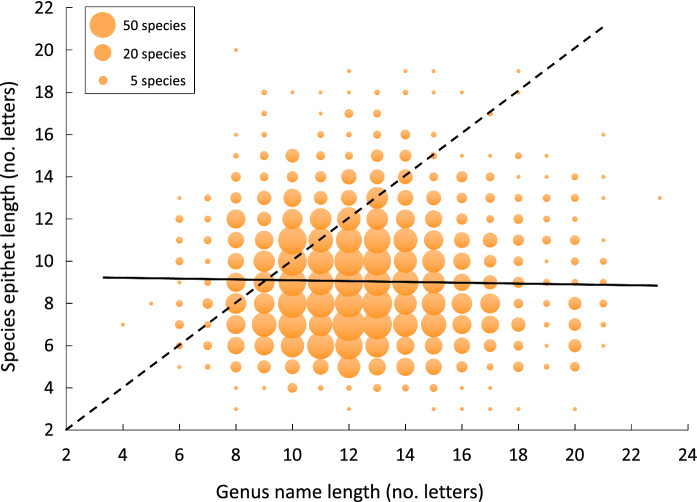
Bubble plot of the length of species epithets as a function of the length of genus names among 3016 species of helminth parasites described between 2000 and 2021 inclusively. The diameter of each bubble is proportional to the number of species with the corresponding genus name and species epithet length values. The solid line is the line of best fit, whereas values along the broken line represent cases where the species epithet and genus name have the exact same length.

Finally, the GLMM found no relationship between the length of species epithets and that of genus names (Table 3). The length of species epithets remains exactly the same on average regardless of the length of the genus name (Fig. 3). However, species epithets are generally shorter than genus names. The species epithet was shorter than the genus name for 2260 species (74.9% of cases), exactly the same length for 238 species (7.9%), and longer for 518 species (17.2%).

## Discussion

The inventory of parasite biodiversity on Earth is far from complete. The number of new parasite species discovered and described every year has been increasing in recent decades, and several hotspots of biodiversity are yet to be fully explored for the parasite species they harbour (Poulin & Presswell 2016; Jorge & Poulin 2018; Carlson et al. 2020). It is therefore a good time to re-examine some of the practices associated with the description of new parasite species, including the choice of species epithets.

Our findings indicate that, on average, the length of species epithets given to newly described helminth species has not changed over the past two decades. However, we found a small but significant taxonomic bias in the length of species epithets: acanthocephalans are generally given epithets slightly longer than those chosen for species in other helminth taxa. The reasons for this difference are unclear, but may simply arise from the personal preferences of the most prolific taxonomists specialising in acanthocephalans. Interestingly, acanthocephalans also tend to have the longest genus names. In our dataset, treating each occurrence of a genus name separately even if they appear multiple times, genus names of acanthocephalans are 14.4 letters long on average compared to 13.4 for monogeneans, 13.0 for cestodes, 12.2 for trematodes, and 11.3 for nematodes. Therefore, acanthocephalans generally have the longest binomial Latin names of all helminths. This should perhaps be taken into consideration when naming new acanthocephalan species in the future.

We also found no evidence of a negative relationship between the length of species epithets and that of genus names. Had we found one, such a relationship would have suggested an attempt, whether conscious or not, to compensate for very long genus names by choosing a short species epithet for new species assigned to such genera. Perhaps such efforts to match long names with short ones should be encouraged in future, to keep the overall binomial name within reasonable length.

Earlier, we used the same dataset to assign species epithets to 5 broad etymological categories, based on the source of inspiration used to name a new species (Poulin et al. 2022). Species were categorised into those named for their morphology, for their host, for their type locality, after an eminent scientist, or for something else. A preliminary look at whether the length of species epithets differed among these categories, or subcategories within the 5 main ones, indicated that they do not (data not shown). Thus, the inspiration behind a species epithet does not influence its length.

The only comparable study we are aware of is an investigation of the names of over 48,000 spider species described since the middle of the 18^th^ century (Mammola et al. 2022). The frequency distribution of the lengths of species epithets among spiders is strikingly similar to ours, with the same shape and mode, resulting in practically the same average length. In addition, there was no temporal change in the average length of species epithets among spiders, even though the spider data set spanned more than two centuries (Mammola et al. 2022), whereas ours covered just over two decades. It seems therefore that the few patterns we observed are not unique to parasite species, but may reflect broader practices in taxonomy.

Previous commentators have provided guidelines for the formation of species names that conform to the grammatical rules of Latin, and/or for the correct usage of species names after they are coined (Sangster & Pope 2000; Notton et al. 2011; Šlapeta 2013; Vendetti & Garland 2019). Based on our findings, we would like to remind all readers of article 25C of the ICZN (https://www.iczn.org/), which states that names should be chosen with their subsequent users in mind, so that they are as much as possible compact, euphonious and memorable. The only absolute requirement for a species epithet is that it must be unique amongst known species within the same genus. We therefore encourage taxonomists to choose species epithets that are no longer than 12–13 letters, which seems the maximum that most biologists would be comfortable with, whatever their native language. Shorter names are not necessarily ‘sweeter’, i.e. more pleasant sounding when spoken out loud, but they are likely easier to pronounce. As is true of scientific jargon in general, simpler is often better.

## Supplementary Information

Below is the link to the electronic supplementary material.

Supplementary file1 (XLSX 282 kb)

## Data Availability

The full dataset is available as Supplementary Material.

## References

[CR1] Amin OM, Heckmann RA, Ali AH (2018). The finding of Pacific transvenid acanthocephalan in the Arabian Gulf, with the description of *Paratrajectura longcementglandatus* n. gen., n. sp. from perciform fishes and emendation of Transvenidae. Journal of Parasitology.

[CR2] Amin OM, Heckmann RA, Van Ha N (2011). Description of two new species of *Rhadinorhynchus* (Acanthocephala, Rhadinorhynchidae) from marine fish in Halong Bay, Vietnam, with a key to species. Acta Parasitologica.

[CR3] Bates, D., Mächler, M., Bolker, B., & Walker, S. (2015) Fitting linear mixed-effects models using lme4. *Journal of Statistical Software 67*, 1–48. 10.18637/jss.v067.i01

[CR4] Bray RA, Cribb TH (2003). Lepocreadiidae (Digenea) from the batfish of the genus *Platax* Cuvier (Teleostei: Ephippidae) from the southern Great Barrier Reef, Queensland, Australia. Systematic Parasitology.

[CR5] Carlson CJ, Dallas TA, Alexander LW, Phelan AL, Phillips AJ (2020). What would it take to describe the global diversity of parasites?. Proceedings of the Royal Society B.

[CR6] Cone D, Abbott C, Gilmore S, Burt M (2010). A new genus and species of gyrodactylid (Monogenea) from silver hake, *Merluccius bilinearis*, in the Bay of Fundy, New Brunswick, Canada. Journal of Parasitology.

[CR7] Corner RD, Cribb TH, Cutmore SC (2020). A new genus of Bucephalidae Poche, 1907 (Trematoda: Digenea) for three new species infecting the yellowtail pike, *Sphyraena obtusata* Cuvier (Sphyraenidae), from Moreton Bay, Queensland, Australia. Systematic Parasitology.

[CR8] Cruces CL, Chero JD, Sáez G, Luque JL (2021). *Bicentenariella* n. g. (Monogenea: Dactylogyridae) including descriptions of three new species and two new combinations from serranid fishes (Actinopterygii: Serranidae: Anthiinae) in the South American Pacific Ocean. Systematic Parasitology.

[CR9] Jorge F, Poulin R (2018). Poor geographical match between the distributions of host diversity and parasite discovery effort. Proceedings of the Royal Society B.

[CR10] Kuzmin Y, Tkach VV, Brooks DR (2007). Two new species of *Rhabdias* (Nematoda: Rhabdiasidae) from the marine toad, *Bufo marinus* (L.) (Lissamphibia: Anura: Bufonidae), in Central America. Journal of Parasitology.

[CR11] Mammola S, Viel N, Amiar D, Mani A, Hervé C, Heard SB, Fontaneto D, Pétillon J (2022). Taxonomic practice, creativity, and fashion: what’s in a spider name?. BioRxiv preprint.

[CR12] Moravec, F. & Justine, J.-L. (2009) New data on dracunculoid nematodes from fishes off New Caledonia, including four new species of *Philometra* (Philometridae) and *Ichthyofilaria* (Guyanemidae). *Folia Parasitologica 56*, 129–142. 10.14411/fp.2009.017 10.14411/fp.2009.017 19606788

[CR13] Moravec F, Justine J-L (2010). Some trichinelloid nematodes from marine fishes off New Caledonia, including description of *Pseudocapillaria novaecaledoniensis* sp. nov. (Capillariidae). Acta Parasitologica.

[CR14] Notton, D., Michel, E., Dale-Skey, N., Nikolaeva, S., & Tracey, S. (2011) Best practice in the use of the scientific names of animals: support for editors of technical journals. *Bulletin of Zoological Nomenclature 68*, 313–322. 10.21805/bzn.v68i4.a15

[CR15] Poulin R, Presswell B (2016). Taxonomic quality of species descriptions varies over time and with the number of authors, but unevenly among parasitic taxa. Systematic Biology.

[CR16] Poulin R, Presswell B (2022). Is parasite taxonomy really in trouble? A quantitative analysis. International Journal for Parasitology.

[CR17] Poulin R, McDougall C, Presswell B (2022). What’s in a name? Taxonomic and gender biases in the etymology of new species names. Proceedings of the Royal Society B.

[CR18] R Core Team (2022). R: a language and environment for statistical computing.

[CR19] Sangster NC, Pope SE (2000). Quid significat nomen? (What’s in a name?). International Journal for Parasitology.

[CR20] Šlapeta, J. (2013) Ten simple rules for describing a new (parasite) species. *International Journal for Parasitology – Parasites and Wildlife 2*, 152–154. 10.1016/j.ijppaw.2013.03.005 10.1016/j.ijppaw.2013.03.005 PMC3862503 24533329

[CR21] Soo OYM, Tan WB (2021). *Hamatopeduncularia* Yamaguti, 1953 (Monogenea: Ancylodiscoididae) from catfish off Peninsular Malaysia: description of two new species and insights on the genus. Parasitology International.

[CR22] Vendetti JE, Garland R (2019). Species name formation for zoologists: a pragmatic approach. Journal of Natural History.

